# Supplementary material for: Association of Left Atrial Size Measured by Non-Contrast Computed Tomography with Cardiovascular Risk Factors—The Danish Cardiovascular Screening Trial (DANCAVAS)
Source: Diagnostics (Basel). 2022 Jan 19;12(2):244. doi: 10.3390/diagnostics12020244 (PMC8871467; doi:10.3390/diagnostics12020244)
Supplement: Supplementary file 1 [file diagnostics-12-00244-s001.zip › diagnostics-1563330-supplementary.pdf]

Table S1. Linear regression of left atrium area index (cm^2/m^2) in men, analyzed in three subgroups depending on heart rate.

	Heart Rate < 65 *n* = 4574		Heart Rate 65–75 *n* = 3017		Heart Rate ≥ 75 *n* = 3133	
**Variable**	**Δ LA area index (95% CI)**	***p* Value**	**Δ LA Area Index (95% CI)**	***p* Value**	**Δ LA Area Index (95% CI)**	***p* Value**
Age (years) *	0.05 (0.03;0.07)	<0.001	0.06 (0.04;0.09)	<0.001	0.06 (0.03;0.08)	<0.001
Smoking						
Former	−0.34 (−0.47; −0.21)	<0.001	−0.44 (−0.61; −0.27)	<0.001	−0.53 (−0.74; −0.33)	<0.001
Current	−0.93 (−1.13; −0.74)	<0.001	−0.83 (−1.06; −0.60)	<0.001	−1.29 (−1.54; −1.04)	<0.001
Pulse pressure (mmHg) *	0.03 (0.02; 0.03)	<0.001	0.02 (0.02; 0.03)	<0.001	0.03 (0.02; 0.03)	<0.001
HbA1c (mmol/mol) *	−0.02 (−0.03; −0.01)	<0.001	−0.02 (−0.03; −0.01)	0.003	−0.01 (−0.02; 0.00)	0.100
Total cholesterol (mmol/L) *	−0.09 (−0.15; −0.03)	0.004	−0.09 (−0.17; −0.13)	0.022	−0.14 (−0.23; −0.06)	0.001

* By 1 unit increment.
